# Ethanol Solvothermal Treatment on Graphitic Carbon Nitride Materials for Enhancing Photocatalytic Hydrogen Evolution Performance

**DOI:** 10.3390/nano12020179

**Published:** 2022-01-06

**Authors:** Phuong Anh Nguyen, Thi Kim Anh Nguyen, Duc Quang Dao, Eun Woo Shin

**Affiliations:** School of Chemical Engineering, University of Ulsan, Daehakro 93, Nam-gu, Ulsan 44610, Korea; anhphuong.nguyen1150@gmail.com (P.A.N.); nguyenthikimanhtb@gmail.com (T.K.A.N.); quangdao.ys@gmail.com (D.Q.D.)

**Keywords:** graphitic carbon nitride, ethanol solvothermal, Pt oxidation state, charge separation

## Abstract

Recently, Pt-loaded graphic carbon nitride (g-C_3_N_4_) materials have attracted great attention as a photocatalyst for hydrogen evolution from water. The simple surface modification of g-C_3_N_4_ by hydrothermal methods improves photocatalytic performance. In this study, ethanol is used as a solvothermal solvent to modify the surface properties of g-C_3_N_4_ for the first time. The g-C_3_N_4_ is thermally treated in ethanol at different temperatures (T = 140 °C, 160 °C, 180 °C, and 220 °C), and the Pt co-catalyst is subsequently deposited on the g-C_3_N_4_ via a photodeposition method. Elemental analysis and XPS O 1s data confirm that the ethanol solvothermal treatment increased the contents of the oxygen-containing functional groups on the g-C_3_N_4_ and were proportional to the treatment temperatures. However, the XPS Pt 4f data show that the Pt^2+^/Pt^0^ value for the Pt/g-C_3_N_4_ treated at ethanol solvothermal temperature of 160 °C (Pt/CN-160) is the highest at 7.03, implying the highest hydrogen production rate of Pt/CN-160 is at 492.3 μmol g^−1^ h^−1^ because the PtO phase is favorable for the water adsorption and hydrogen desorption in the hydrogen evolution process. In addition, the electrochemical impedance spectroscopy data and the photoluminescence spectra emission peak intensify reflect that the Pt/CN-160 had a more efficient charge separation process that also enhanced the photocatalytic activity.

## 1. Introduction

Owing to the abundance and renewability of sunlight and water, a solar-driven water-splitting process using photocatalysts is considered a long-term sustainable technology for producing hydrogen—a clean and renewable energy source. Even though various photocatalysts, such as TiO_2_, SrTiO_3_, CdS, TaON, ZrO_2_, and their assemblies, have been extensively studied, the development of novel photocatalysts that significantly enhance hydrogen production performance is still a challenging research area [1,2,3,4,5].

Graphitic carbon nitride (g-C_3_N_4_), a metal-free polymeric semiconductor, has attracted much interest because of its low cost, non-toxicity, ease of preparation, high physicochemical stability, and suitable band gap energy (2.7 eV) for visible light utilization [6], since the first introduction of its photocatalytic activity toward hydrogen evolution in 2009. Nonetheless, the photocatalytic application of bulk g-C_3_N_4_ (BCN) is restricted by some disadvantages, such as a low specific surface area and the rapid recombination rate of charge carriers. To tackle these challenges, a great deal of effort has been made by researchers over the past few years, including metal or non-metal doping [7,8,9], liquid-phase exfoliation [10], chemical oxidation [11,12,13,14], and heterojunction fabrication [15,16,17,18]. Despite the positive outcomes of these methods, limitations remain; they are time- and cost-consuming methods that require a toxic chemical involvement, they are multi-step, and they require complicated synthetic procedures. According to the literature, the hydrothermal method is an effective modification strategy to introduce O-containing functional groups into the g-C_3_N_4_, which has been known to exert a desirable effect on photocatalytic hydrogen evolution [12,19,20,21]. Unfortunately, the hydrothermal modification struggles to optimize the morphological and chemical structure of the g-C_3_N_4_, in which water can act as a strong solvent and destroy the crystalline structure. Thus, the morphological instability in the hydrothermally modified, g-C_3_N_4_-suppressed, photoinduced electron–hole pair separation and widened the band gap, resulting in a decrease in the photocatalytic activity for hydrogen production [20,21].

Herein, ethanol is used as a novel solvent for a simple and environmentally friendly solvothermal treatment to modify g-C_3_N_4_. The solvothermal temperatures varied from 140 °C to 220 °C to adjust the solvothermal modification extent. The effects of the ethanol solvent on the physicochemical and optical properties of the photocatalysts and the photocatalytic performance are investigated to find the essential properties to determine the photocatalytic activity. Compared with water in the hydrothermal method, ethanol proves to be a weaker but more effective agent to introduce the O-containing functional groups onto the g-C_3_N_4_ photocatalysts. Interestingly, the highest Pt^2+^ species are found for Pt/g-C_3_N_4_ treated at 160 °C, inhibiting the unfavorable H_2_ backward oxidation reaction and resulting in the superior photocatalytic performance toward hydrogen evolution under visible light irradiation. In addition, the Pt/CN-160 photocatalyst facilitates the photoinduced charge carrier separation, which is illustrated by the electrochemical impedance spectroscopy (EIS) and photoluminescence (PL) spectra.

## 2. Materials and Methods

### 2.1. Chemicals

All chemicals in the experiments were used without further purification. All catalyst syntheses and hydrogen evolution reactions were performed with deionized (DI) water. Thiourea (CH_4_N_2_S, ≥99%), triethanolamine (TEOA, C_6_H_15_NO_3_, 99%), and chloroplatinic acid hexahydrate (H_2_PtCl_6_.6H_2_O) were purchased from Sigma-Aldrich (Gyeonggi, Korea). Ethyl alcohol (C_2_H_5_OH, 99.9%) was obtained from Daejung Chemicals and Metals Co., Ltd. (Gyeonggi, Korea).

### 2.2. Preparation of Ethanol Solvothermal-Treated g-C_3_N_4_

It has been reported that the presence of foreign atoms in precursors (e.g., sulfur) positively affect the optical and electronic properties of bulk g-C_3_N_4_ in photocatalytic performance [13,22]. Therefore, thiourea was chosen to produce bulk g-C_3_N_4_ in this work. Briefly, thiourea was directly heated at 550 °C with a ramping rate of 5 °C/min for 4 h in air. The resulting product was ground up to obtain bulk g-C_3_N_4_.

The ethanol-treated g-C_3_N_4_ catalysts were synthesized via a solvothermal process. In detail, a certain amount of the obtained bulk g-C_3_N_4_ (1.0 g) was dissolved into an ultrasonic beaker containing 100 mL of C_2_H_5_OH solution before sonicating for 2 h with a bath temperature of 30 °C. After that, the mixture was poured into a Teflon-lined autoclave (150 mL internal volume) that was then sealed and heated in an oven for 6 h at a heating rate of 2 °C/min to a specific temperature (140 °C, 160 °C, 180 °C, and 220 °C). After cooling down to room temperature, the resultant products were sequentially centrifugated and washed thoroughly with DI water several times. The samples were designated as BCN, CN-140, CN-160, CN-180, and CN-220 corresponding to the bulk g-C_3_N_4,_ and the solvothermal temperatures of 140 °C, 160 °C, 180 °C, and 220 °C, respectively.

### 2.3. Preparation of Pt/CN Photocatalysts

A photodeposition method was utilized to prepare the Pt-loaded g-C_3_N_4_ samples. Fifty milligrams of CN was suspended in 100 mL of DI water using a magnetic stirrer for 20 min under the inert atmosphere of argon (Ar). Pt (3 wt%) was decorated onto the CN samples using hexachloroplatinic acid (H_2_PtCl_6_.6H_2_O) as a Pt precursor that was dispersed into the prepared CN solution for 20 min. Then, the final solution was photoirradiated by solar-simulated irradiation with continuous magnetic stirring for 60 min. Eventually, the final solid product was obtained by centrifugation, washed with DI water, and dried under a vacuum at −80 °C. The Pt/CN samples were ground into a powder and collected for further characterization.

### 2.4. Characterization

All sample images were obtained via field emission (FE) scanning electron microscopy (SEM; JSM-6500 JEOL, Tokyo, Japan). In addition, the SEM images and the corresponding elemental mappings were collected via a second instrument, energy-dispersive X-ray spectroscopy (EDS; TESCAN MIRA3, Kohoutovice, Czech Republic). The inductively coupled plasma-optical emission spectrometry (ICP-OES) was conducted on a 700-ES spectrometer (Varian, Mulgrave, Australia) to analyze the Pt element contents. The high-resolution transmission microscopy (HR-TEM) images were obtained by a JEL-2100F JEOL instrument (Tokyo, Japan). X-ray diffraction (XRD) was performed on a Rigaku D/MAZX 2500 V/PC high-power diffractometer (Tokyo, Japan) at a scan rate of 2° min^−1^ with a Cu Kα radiation to analyze the crystallinity of the as-prepared samples. The distances d_(100)_ and d_(002)_ were calculated based on the Bragg’s law d = nλ/2sin θ, where n is the order of reflection (n = 1), λ is the wavelength of the incident X-ray (λ = 0.15415 Å), and θ is the reflection angle. Fourier transform infrared spectra (FTIR) were conducted to examine the presence of functional groups on all samples, using a Nicolet 380, Thermo Scientific Nicolet iS5 instrument (Waltham, MA, USA). To characterize the obtained samples’ chemical composition and electronic structure, X-ray photoelectron spectroscopy (XPS) analysis was performed with a Thermo Scientific Kα X-ray source (Waltham, MA, USA). Elemental analysis (EA) was conducted on a Flash 2000 instrument (Thermal Fisher Scientific, Waltham, MA, USA). The optical properties were collected via ultraviolet–visible (UV–Vis) diffuse reflectance using an SPE-CORD 210 Plus spectroscope (Analytik Jena, Jena, Germany) and PL spectra (Agilent Cary Eclipse fluorescence spectrophotometer, Santa Clara, CA, USA). The charge carrier separation and transfer efficiency were determined with EIS via a VSP BioLogic Science instrument (Seyssinet-Pariset, France) within 0.01–100 kHz frequency range at a +0.7 VSCE direct current potential and a 10 mV AC amplitude. Ten microliters of the sample was loaded onto a 6 mm glassy carbon electrode, known as a working electrode. The electrolyte for the three-electrode system (Ag/AgCl electrode as a reference electrode and Pt wire as a counter electrode) was 1 M NaOH solution.

### 2.5. Photocatalytic H_2_ Evolution

Photocatalytic H_2_ evolution experiments were operated using a class ABA LED solar simulator with a 1.0 SUN output power, corresponding to 100 mW/cm^2^. Initially, 50 mg of CN was dissolved in 90 mL of DI water in a 300 mL internal volume quartz flask via magnetic stirring for 20 min. Then, the H_2_PtCl_6_.6H_2_O solution (3 wt% Pt) was added. After magnetic stirring for 20 min, the Pt co-catalyst was photodeposited in situ under solar irradiation for 60 min. Finally, the hydrogen evolution reaction was performed after 20 min of adding 10 mL of TEOA as a sacrificial agent. The H_2_ product was analyzed by a gas chromatograph (Acme 6100) with a thermal conductivity detector. All reactions were accomplished under an inert atmosphere of Ar at room temperature.

## 3. Results and Discussion

### 3.1. Structural and Chemical Properties

**Figure 1:**
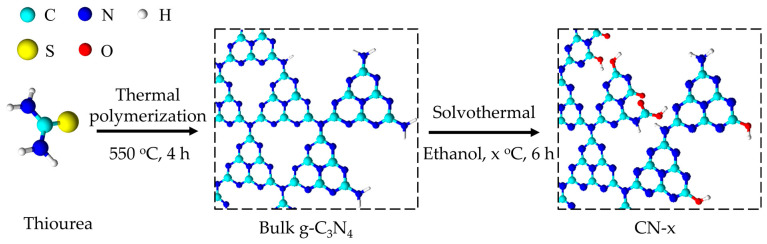
Schematic illustration of the synthetic procedure of ethanol solvothermal-treated CN.

The CN samples modified by the O-containing functional groups were synthesized via a solvothermal process using ethanol as a solvent. The scheme illustrated in Figure 1 shows two main steps, as follows: (1) the thermal polymerization of thiourea at 550 °C in air for 4 h to obtain bulk the g-C_3_N_4_; (2) the post-solvothermal treatment using ethanol solvent at different temperatures.

**Figure 2:**
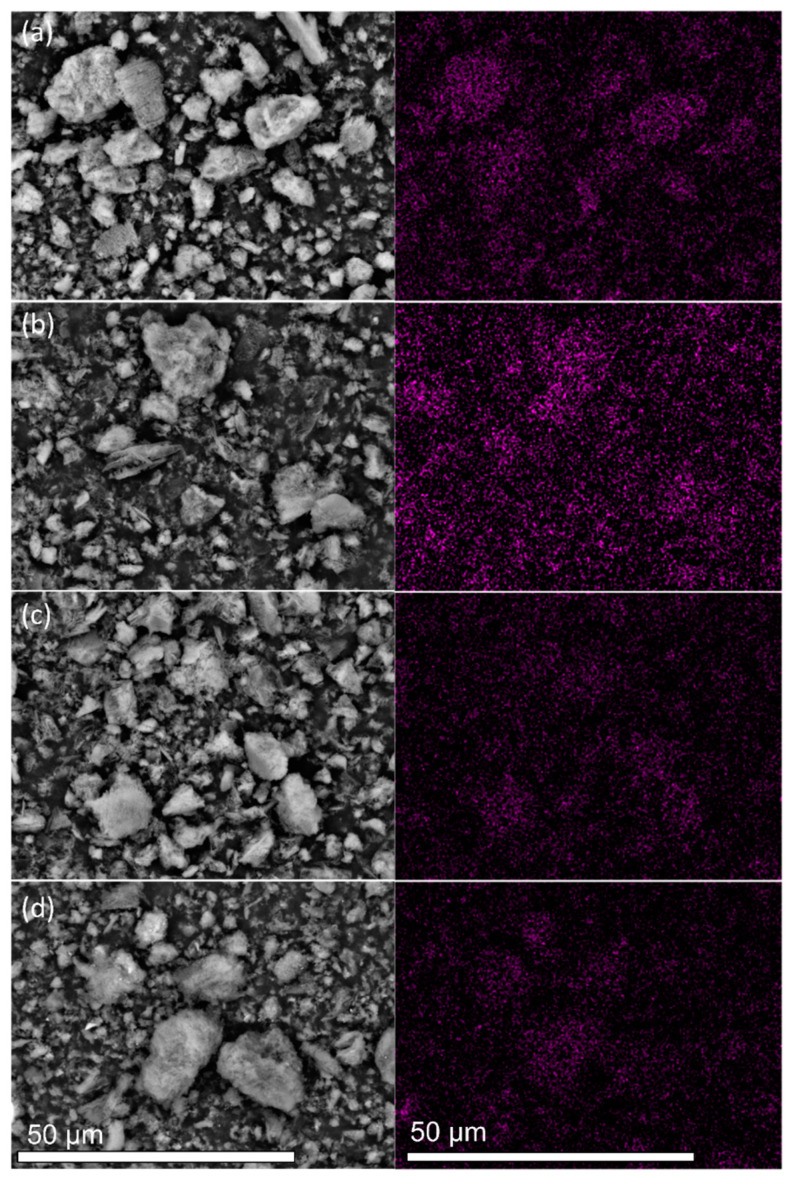
SEM images and the corresponding EDS Pt elemental mappings of the (**a**) Pt/CN-140, (**b**) Pt/CN-160, (**c**) Pt/CN-180, and (**d**) Pt/CN-220 photocatalysts.

**Figure 3:**
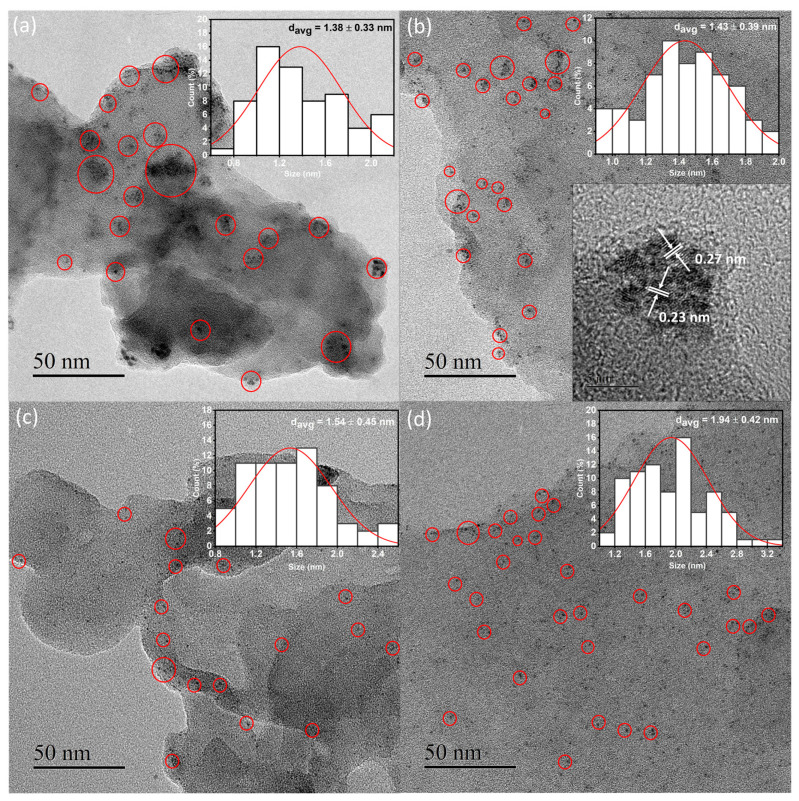
TEM images of the Pt/CN-140 (**a**), Pt/CN-160 (**b**), Pt/CN-180 (**c**), and Pt/CN-220 (**d**) photocatalysts. The insets of (**a**–**d**) are the size distribution of the Pt particles. Another inset in panel (**b**) is the corresponding HR-TEM image of the Pt/CN-160 photocatalysts.

The TEM and SEM analyses characterized the morphological and textural properties of all samples. The FE-SEM images of the samples display the characteristic sheet-like morphological structure (Figure S1). The EDS elemental mappings in Figure 2 and Figure S2 depict the distributions of C, N, and Pt in the four samples. Although similar amounts of Pt were loaded onto the four samples (Table 1), the purple spots assigned to Pt were more homogeneously dispersed in the Pt/CN-160 sample than were those in the Pt/CN-140, Pt/CN-180, and Pt/CN-220 (Figure 2) samples. The TEM images in Figure 3 also demonstrate that the Pt species observed in all four samples was reduced from Pt^4+^ during the photodeposition process. The areas marked by red circles correspond to Pt and PtO particles or clusters anchoring on the g-C_3_N_4_ surface. These particles are nearly spherical with an order of less than 2 nm in average size. Furthermore, it is evident that, although the average diameter of a Pt particle in Pt/CN-140 is the smallest at 1.38 ± 0.33 nm, some large Pt clusters are still found, implying an agglomeration of Pt species during the photodeposition process. Pt/CN-160, by contrast, possesses a more uniform Pt dispersion at an average particle size of 1.43 ± 0.39 nm. In the inset of Figure 3b, the high-resolution TEM image of the Pt/CN-160 reveals the lattice fringes of 0.23 and 0.27 nm that can be attributed to the (111) plane of Pt and the (002) face of PtO, respectively [23,24]. This can be connected with further support from the Pt 4f XPS results in which PtO is one of the main Pt species after the photodeposition process.

**Figure 4:**
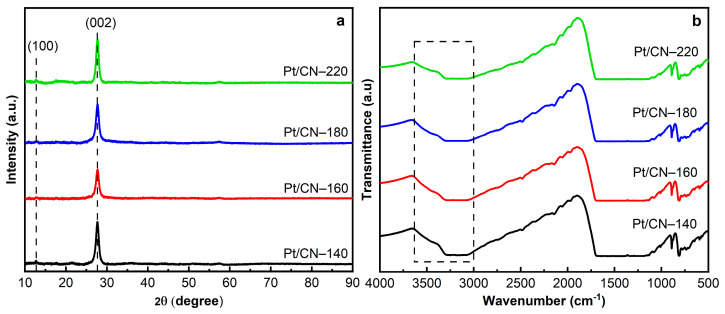
The XRD patterns (**a**) and FTIR spectra (**b**) of the Pt/CN photocatalysts.

Figure 4a depicts the XRD patterns of the Pt/g-C_3_N_4_ after the different solvothermal temperatures. Two characteristic diffraction peaks at 27.6° and 12.7°, which can be ascribed to the interlayer (002) and intralayer (100) planes of the g-C_3_N_4_, respectively, clearly reflect the existence of graphitic-like layer structures in all the samples [25,26]. Bragg’s law showed no significant change in the d-spacing values among the four samples, implying the robust solvothermal stability of the g-C_3_N_4_ photocatalysts (even those prepared with the ethanol solvent) (Table 1) [25,27,28]. As is shown in Figure 4a, the interlayer stacking (002) peak intensity of Pt/CN-160 is lower than that of the other samples, likely indicating that there would be a loss in the stacking ordered structure of the g-C_3_N_4_ nanosheet, resulting in a little-layered structure of g-C_3_N_4_ [7,29,30,31,32]. However, when the solvothermal temperature is above 180 °C, the (002) peaks become stronger; this is likely associated with the enhanced crystallinity since defects can be recovered by re-polymerization during the solvothermal process [32].

To investigate the formation of oxygen-containing functional groups on g-C_3_N_4_, FTIR, XPS, and EA were performed. Figure 4b shows the FTIR spectra of the Pt/g-C_3_N_4_ samples at different treatment temperatures. All FTIR spectra represent the typical peaks at 810 and 1200–1700 cm^−1^ that are attributed to the breathing mode of tri-s-triazine units and the stretching vibration modes of aromatic C–N and C = N heterocyclics, respectively [20,33,34,35]. Another peak appearing at 888 cm^−1^ originates from the deformation mode of N–H bonding [33,36]. Notably, in the broad band between 3000 and 3600 cm^−1^, owing to the combination of the N–H and O–H stretching vibrations, the O–H bands become relatively stronger with the increase in solvothermal treatment temperature, indicating the introduction of –OH functional groups [12,33,37]. This is consistent with the XPS data shown below.

**Figure 5:**
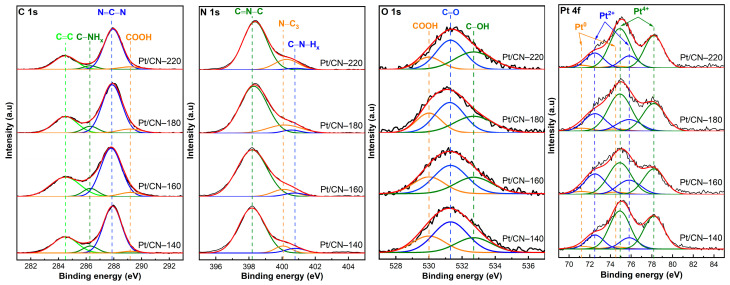
The XPS spectra of C 1s, N 1s, O 1s, and Pt 4f of the Pt/CN photocatalysts.

The XPS analysis was conducted to identify the elements’ specific bonding and chemical states. The survey spectra of all the samples are shown in Figure S3, containing sharp peaks at about 74, 287, 398, and 532 eV, corresponding to Pt 4f, C 1s, N 1s, and O 1s, respectively [19,24]. Figure 5 shows the typical C 1s spectra of the four samples. These could be deconvoluted into three different peaks appearing at the binding energies of 284.5, 286.3, and 287.8, which correspond to adventitious aliphatic carbon atoms (C–C), the sp^3^ C atoms (C–NH_x_), and the sp^2^ C species (N = C–N), respectively [7,12,15,19,29,36,38]. Moreover, an additional peak at 289.2 eV arises from the –COOH species generated during the solvothermal process [20]. Notably, the four samples’ C–NH_x_ peaks detected at 286.3 eV are gradually weakened because of the missing –NH_2_ groups after the solvothermal treatment [29]. This result is also supported by the high-resolution XPS spectra of N 1s. As depicted in Figure 5, the spectra of the four samples could be fitted into the three peaks detected at approximately 398.2, 400.1, and 400.7 eV. The peak at 400.1 eV assigned to a tertiary N atom (N_3C_) and the dominant peak at around 398.2 eV could be attributed to the sp^2^-hybridized N bonded to two C atoms (C = N–C, N_2C_) [7,12,15,20,25,29,34,36,37,39]. As the solvothermal temperature increases, these N_2C_ and N_3C_ peaks shift from 398.2 and 400.1 eV in Pt/CN-140 to the higher binding energies of 398.4 and 400.3 eV in the Pt/CN-220 sample, respectively. This is likely due to the replacement of the amino groups by hydroxyl groups [26,40]. In addition, the weak peak located at 400.7 eV is related to amino functional groups (C–NH_x_) derived from the incomplete condensation of heptazine structures [12,15,29,34]. Particularly, in Table 2, at the mild solvothermal treatment condition (140 °C), the percentage of C–N–H_x_ is the highest at 6.79 atomic percentage (at%). On the contrary, those of Pt/CN-160 and Pt/CN-180 gradually decrease to 4.29 and 3.48 wt%, respectively, and the Pt/CN-220 sample finally reaches the lowest value at 1.86 wt%. Moreover, the proportions of N atoms (wt%), calculated from the EA, as well as the relative peak area ratio of N_2C_/N_3C_, also decreased gradually, suggesting the loss of lattice N_2C_ atoms through the solvothermal process and further confirming the formation of nitrogen defects and O-containing functional groups [12,26,41].

The existence of carbonyl (C=O), carboxyl (–COOH), and hydroxyl groups (–OH) was confirmed by the O 1s XPS spectra. In Figure 5, the high-resolution O 1s spectra of the four samples exhibit similar peaks centered at 530.0, 531.3, and 532.7 eV that can be assigned to –COOH, C=O, and –OH functional groups, respectively [21,37,42,43]. The atomic percentage of –OH groups is illustrated in Table 2. The C–OH group percentage increases considerably from 25.03 wt% in Pt/CN-140 to 36.54 wt% in the Pt/CN-220 sample. To support the presence of the O-containing functional groups, EA was conducted (Table 2). The Pt/CN-140 sample contains 4.07 wt% of the proportion of O atoms, while that of the Pt/CN-220 is relatively higher at 5.81 wt%. These results indicate the introduction of the O-containing functional groups from nitrogen-defective sites under the more severe solvothermal treatment condition, in accordance with the C 1s and N 1s results discussed above.

Figure 5 presents the high-resolution XPS data for the four samples in the Pt 4f binding energy peaks, including Pt 4f_5/2_ and Pt 4f_7/2_, in agreement with the spin–orbit splitting of 3.3 eV. Three pairs of doublets were obtained by deconvolution. The sub-peaks at 71.2 and 74.5 eV were assigned to Pt 4f_7/2_ and Pt 4f_5/2_ of metallic Pt^0^, respectively [24,44,45,46]. Meanwhile, Pt^2+^ is represented by the doublet at binding energies of 72.5 and 75.8 eV, indicating that the Pt^0^ and Pt^2+^ species co-exist [12,24,44,45,47,48]. In addition, Pt^4+^ species corresponding to peaks at 74.9 and 78.2 eV are also noted [12]. This large portion of Pt^4+^ results from the partly unreduced H_2_PtCl_6_.6H_2_O precursor in the photodeposition process. From the data list in Table 2, the Pt^4+^ percentage of Pt/CN-160 sample is 59.33 wt%, lower than those of other samples (over 65 wt%), implying that the larger amounts of Pt^2+^ and Pt^0^ species are deposited onto the surface of g-C_3_N_4_*,* which has been treated at the solvothermal temperature of 160 °C. This result is in accordance with the EDS Pt mappings result mentioned above. The estimated Pt^2+^/Pt^0^ ratio using the relative peak areas was the highest in the Pt/CN-160 sample, at 7.03 (Table 2). In other words, most of the reduced Pt species in Pt/CN-160 were positively charged (Pt^2+^), which is beneficial to the hydrogen evolution reaction. It has been reported that Pt^2+^ improves the hydrogen evolution rate by facilitating the photoinduced charge separation and inhibiting the undesirable hydrogen backward oxidation reaction [12,35,44,47].

### 3.2. Optical and Photoelectrochemical Properties

**Figure 6:**
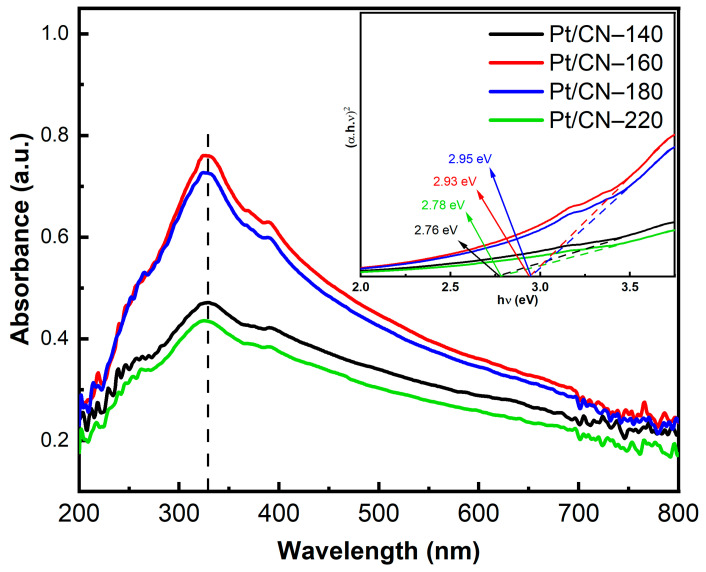
The UV–Vis spectra of the Pt/CN photocatalysts (inset: the corresponding Tauc plots).

These modified samples’ light absorption and band structure properties were investigated using UV–Vis absorption spectroscopy, as is depicted in Figure 6. The absorption spectra of all the as-synthesized samples reach the maximum peaks in the ultraviolet range (wavelengths around 325 nm) with shoulders extending to the visible light region, indicating that all the samples are able to harvest photons from visible light utilizing the O-containing functional groups. When elevating the solvothermal temperature, the absorbance value underwent slight blueshifts, likely because of the well-known quantum confinement effect and the introduction of O-containing functional groups [12,20,32]. Furthermore, Pt/CN-160 and Pt/CN-180 samples reflect higher absorbance intensities compared with those of Pt/CN-140 and Pt/CN-220, indicating that the formers exhibit the stronger absorption ability to harvest more photons under the same irradiation condition. The corresponding band gap values were estimated based on the fitting of Tauc plots (Figure 6) are listed in Table 1. The calculated band gaps of Pt/CN-140, Pt/CN-160, Pt/CN-180, and Pt/CN-220 are 2.76, 2.93, 2.95, and 2.78 eV, respectively. Despite the widened band gap of the Pt/CN-160, this catalyst is still able to exhibit the strongest visible light absorption ability due to the highest absorbance intensity among all four samples.

**Figure 7:**
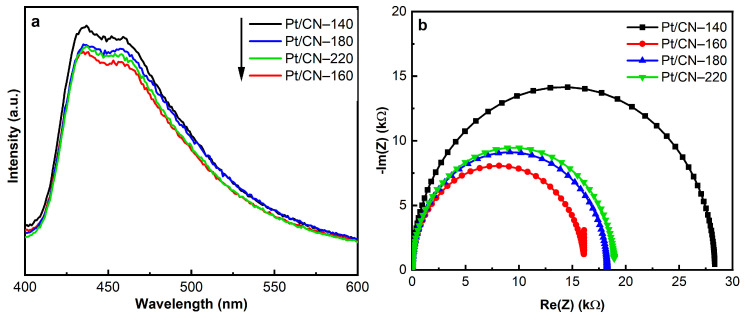
The PL spectra (**a**) and EIS Nyquist plots (**b**) of the Pt/CN photocatalysts.

The separation and transfer ability of the photogenerated charge carriers were inspected using PL and EIS analysis. The PL spectra of the as-prepared samples, shown in Figure 7a, represent the strong characteristic peaks around 435 nm that can be assigned to the band-to-band recombination of photoinduced electrons and holes. Notably, the Pt/CN-160 sample exhibits a quenched PL, implying the efficient suppression of the photogenerated charge carriers’ recombination compared with the Pt/CN-140, Pt/CN-180, and Pt/CN-220 photocatalysts. As is shown in Figure 7b, the EIS spectra provide a similar trend to the above PL results. A comparison of the Pt/CN-160 EIS spectrum with those of its counterparts demonstrates that the former exhibits a smaller arc radius on the Nyquist plots, indicating its lower charge transfer resistance and the most effective photoinduced electron–hole pairs separation. This possibly results from the addition of O-containing functional groups that can act as electron acceptors and increase the thickness of the depletion region, hence improving the charge separation efficiency [49,50,51]. Another possible reason for this enhancement is the high work function of Pt^2+^, which has been in favor of the electron-withdrawing process, accelerating the electron transfer ability and enhancing the hydrogen evolution performance [46,48,52].

### 3.3. Photocatalytic Activity

**Figure 8:**
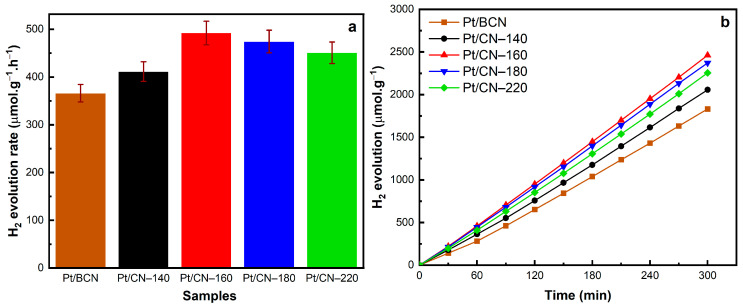
The H_2_ evolution rates (**a**) and photocatalytic H_2_ evolution results over time (**b**) of the Pt/BCN and Pt/CN photocatalysts.

The results discussed above agree with those of the hydrogen evolution performance shown in Figure 8. The photocatalytic hydrogen production experiments of solvothermal-treated photocatalysts were performed under simulated solar irradiation using 10 vol% of TEOA as the sacrificial agent and 3 wt% Pt as the co-catalyst. In comparison with Pt/BCN, all the treated samples exhibit an improvement in the hydrogen evolution rate of over 400 μmol g^−1^ h^−1^ (Figure 8a). Among them, Pt/CN-160 shows an optimal performance, generating up to 2461.7 μmol g^−1^ H_2_ in 5 h with linear growth (Figure 8b), corresponding to 492.3 μmol g^−1^ h^−1^ of H_2_ production rate (Figure 8a). The enhanced photocatalytic activity resulted from generating the optimized amounts of O-containing functional groups via the solvothermal treatment and Pt^2+^ species during the photodeposition process, both of which play pivotal roles in facilitating the separation of photoinduced charge carriers and promoting surface hydrogen evolution reaction.

## 4. Conclusions

In this study, a facile solvothermal method of modifying the g-C_3_N_4_ electronic structure and the oxidation state of the Pt co-catalyst was developed for application to the hydrogen evolution reaction. During the solvothermal process, an ethanol solvent introduced the O-containing functional groups onto the surface of CN, resulting in the formation of photodeposited Pt^2+^ species. This promoted the electron transfer ability and suppressed the back-oxidation reaction of H_2_ during the water-splitting reaction. For these reasons, the Pt/CN-160 catalyst, with the best performance in charge carrier separation and the highest portion of Pt^2+^, exhibited photocatalytic activity of 492.3 µmol g^−1^ h^−1^—the best of all the samples. This study may provide an environmentally friendly and practical approach for preparing graphitic carbon nitride-based photocatalysts for hydrogen evolution reactions.

## Figures and Tables

**Table 1:** Physicochemical and band gap values of the Pt/CN photocatalysts.

Sample	Pt Content (wt%) ^a^	d_(100)_ (nm) ^b^	d_(002)_ (nm) ^b^	Band Gap (eV) ^c^
Pt/CN-140	2.66	0.70	0.3224	2.76
Pt/CN-160	2.62	0.69	0.3220	2.93
Pt/CN-180	2.74	0.70	0.3222	2.95
Pt/CN-220	2.10	0.69	0.3221	2.78

^a^ Obtained from ICP measurement. ^b^ Calculated according to Bragg’s law in XRD analysis. ^c^ Estimated from Tauc plots in UV–Vis analysis.

**Table 2:** Elemental analysis and C, N, and Pt phases of the Pt/CN photocatalysts.

Sample	Content (wt%) ^a^			C-OH ^b^ (at%)	C–N–H_x_ ^c^ (at%)	N_2C_/N_3C_ ^c^	Pt^4+ d^ (at%)	Pt^2+^/Pt^0 d^
	O	H	N					
Pt/CN-140	4.07	1.31	60.91	25.03	6.79	10.40	72.18	4.93
Pt/CN-160	4.96	1.31	60.61	29.30	4.29	8.68	59.33	7.03
Pt/CN-180	5.03	1.28	60.10	31.54	3.48	4.72	65.71	5.99
Pt/CN-220	5.81	1.31	58.53	36.54	1.86	4.68	71.72	5.82

^a^ Obtained from elemental analysis. ^b^, ^c^, ^d^ Determined by C 1s, N 1s, and Pt 4f spectra in XPS analysis, respectively.

## Data Availability

The data presented in this study are available on request from the correspoding author.

## Supplementary Materials

The following supporting information can be downloaded at: https://www.mdpi.com/article/10.3390/nano12020179/s1. Figure S1: FE-SEM images of (a) Pt/CN-140, (b) Pt/CN-160, (c) Pt/CN-180, and (d) Pt/CN-220 photocatalysts, Figure S2: SEM images of (a) Pt/CN-140, (b) Pt/CN-160, (c) Pt/CN-180, and (d) Pt/CN-220 photocatalysts and the corresponding EDS elemental mappings of C, and N, Figure S3: XPS survey spectra of Pt/CN photocatalysts.
