# Supplementary material for: Ethanol Solvothermal Treatment on Graphitic Carbon Nitride Materials for Enhancing Photocatalytic Hydrogen Evolution Performance
Source: Nanomaterials (Basel). 2022 Jan 6;12(2):179. doi: 10.3390/nano12020179 (PMC8779218; doi:10.3390/nano12020179)
Supplement: Supplementary file 1 [file nanomaterials-12-00179-s001.zip › nanomaterials-1535611-supplementary.pdf]

nanomaterials

Supplementary Materials

Ethanol Solvothermal Treatment on Graphitic Carbon Nitride Materials for Enhancing Photocatalytic Hydrogen Evolution Performance

Phuong Anh Nguyen, Thi Kim Anh Nguyen, Duc Quang Dao and Eun Woo Shin *

School of Chemical Engineering, University of Ulsan, Daehakro 93, Nam-gu, Ulsan 44610, Korea; anhphuong.nguyen1150@gmail.com (P.A.N.); nguyenthikimanhtb@gmail.com (T.K.A.N.); quangdao.ys@gmail.com (D.Q.D.)

* Correspondence: ewshin@ulsan.ac.kr; Tel.: +82-52-259-2253

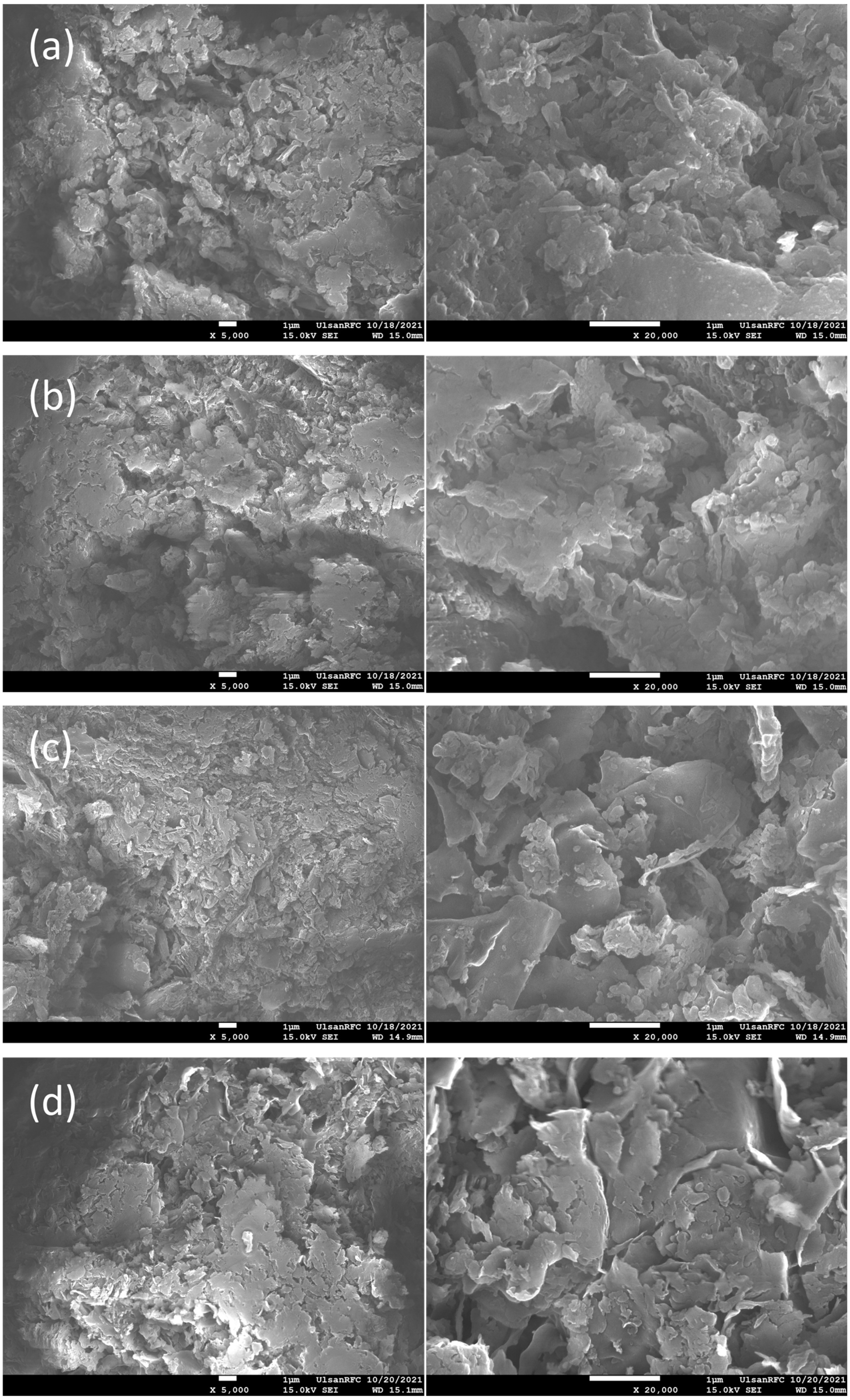

Figure S1. FE-SEM images of (**a**) Pt/CN-140, (**b**) Pt/CN-160, (**c**) Pt/CN-180, and (**d**) Pt/CN-220 photocatalysts.

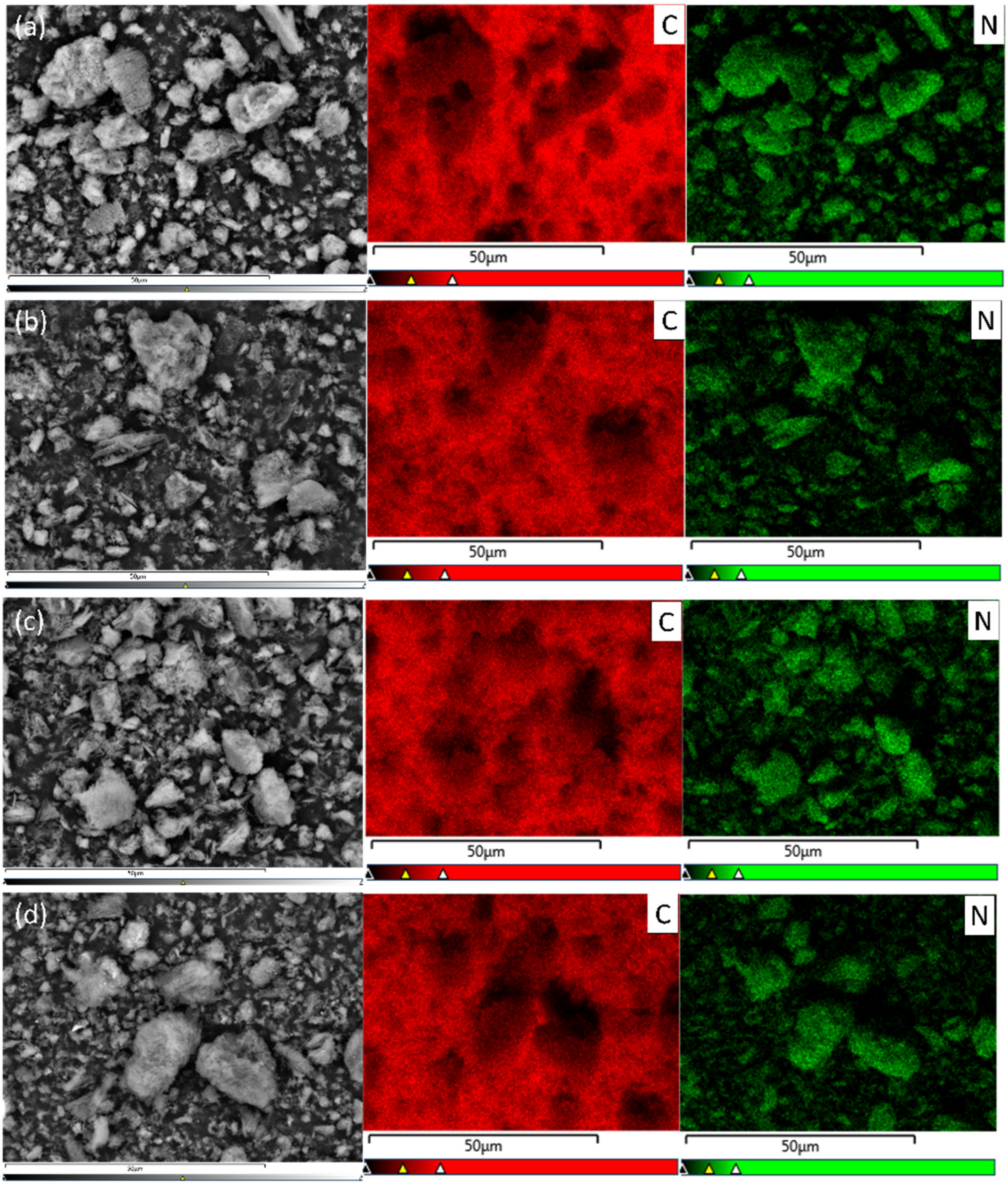

Figure S2. SEM images of (**a**) Pt/CN-140, (**b**) Pt/CN-160, (**c**) Pt/CN-180, and (**d**) Pt/CN-220 photocatalysts and the corresponding EDS elemental mappings of C, and N.

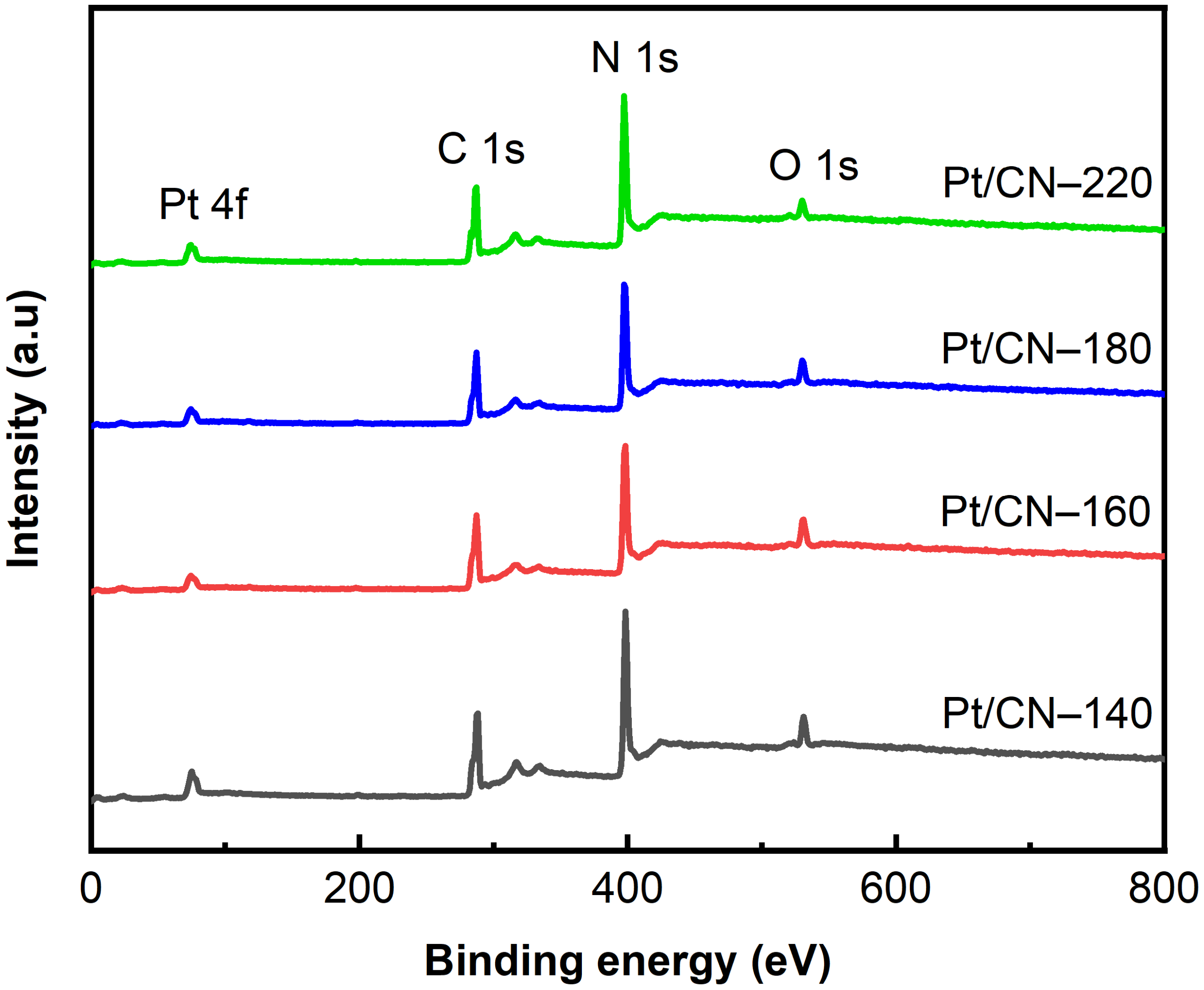

Figure S3. XPS survey spectra of Pt/CN photocatalysts.
